# Calcitonin gene-related peptide as a neuroimmune amplifier in allergic rhinitis: from disease worsening to targeted drug intervention

**DOI:** 10.3389/fimmu.2026.1910048

**Published:** 2026-08-17

**Authors:** Yizuo Qubi, Yingyun Zhou, Hui Wang, Yuaner Mao, Bowen Qiu, Zihao Liu, Xiaoxuan Du, Ying Xu, Minman Wu

**Affiliations:** Yunnan University of Traditional Chinese Medicine, Kunming, China

**Keywords:** allergic rhinitis, CGRP, CGRP receptor antagonist, nasal congestion, neuroimmune, trigeminal sensory nerve

## Abstract

Allergic rhinitis (AR) has long been viewed as a type 2 inflammatory disease driven by IgE, mast cells and eosinophils. However, this classic model does not explain all clinical variation. Some patients continue to experience nasal congestion, nasal hyperreactivity and symptoms triggered by non-specific stimuli despite antihistamines, intranasal corticosteroids or allergen immunotherapy. Calcitonin gene-related peptide (CGRP), a potent vasodilatory neuropeptide mainly released by trigeminal sensory nerves, provides a plausible link between sensory activation, vascular responses, epithelial barrier function and immune-cell regulation. Here, we propose CGRP as a candidate neuroimmune amplifier in AR, while emphasizing that this concept remains a testable framework rather than an established causal pathway. The strongest AR-relevant evidence supports CGRP-mediated nasal vasodilation and obstruction. Evidence that CGRP directly amplifies human type 2 inflammation is more indirect and is partly extrapolated from airway, skin, gut and *in vitro* immune models. Current evidence mainly comes from nasal allergen challenge, neuropeptide measurement in nasal secretions, nasal mucosal tissue studies and intranasal CGRP challenge. CGRP biology is also context-dependent. In some settings, CGRP can suppress dendritic-cell activation or reshape T-cell and ILC2 responses, indicating that it should not be regarded as uniformly pro-inflammatory. Based on the success of CGRP-targeted drugs in migraine, local CGRP receptor antagonists may be useful tools to test a neurogenic AR endotype. These agents demonstrate pharmacological targetability, but not therapeutic efficacy in AR. Future studies should combine standardized nasal-secretion CGRP measurements with congestion-dominant symptoms, nasal hyperreactivity and objective airflow endpoints to determine whether a reproducible CGRP-high AR subgroup exists.

## 1 Introduction: CGRP as a neuroimmune amplifier in AR

Allergic rhinitis (AR) is a common chronic inflammatory disease of the nasal mucosa. Its typical symptoms include nasal itching, sneezing, rhinorrhea and nasal congestion. It may also cause eye symptoms, sleep problems and reduced work or study performance. Because AR is common, recurrent and often linked to asthma, it is an important public health burden (1–4). Current treatment mainly includes second-generation antihistamines, intranasal corticosteroids, leukotriene receptor antagonists, anticholinergic drugs and allergen immunotherapy (AIT) (2, 3). These treatments control inflammation and symptoms in many patients. Yet they do not fully solve residual nasal congestion, non-specific stimulus sensitivity, poor treatment response or chronic nasal hyperreactivity. Thus, the classic IgE-mast cell-eosinophil axis explains the main immune process in AR. It does not explain the full clinical picture.

Recent work on barrier tissue immunity has added sensory nerves to the disease model of allergic disease. Sensory nerves do not only send passive afferent signals. In the nasal mucosa, they are exposed to allergens, pollutants and temperature change. They can act as real-time regulators of inflammation (5, 6). Trigeminal sensory nerves release CGRP, substance P (SP) and vasoactive intestinal peptide (VIP). These neuropeptides can act on blood vessels, epithelium, mast cells, dendritic cells, ILC2s and eosinophils. In this view, AR is not only mucosal type 2 inflammation. It is also a neuroimmune disease at the nasal barrier (7, 8).

Calcitonin gene-related peptide (CGRP) is a central mediator in this network. It is mainly released by sensory neurons, acts through the CLR/RAMP1/RCP receptor complex and activates cAMP-dependent signaling pathways (9). Anti-CGRP and anti-CGRP receptor drugs have been successful in migraine, demonstrating that this pathway is pharmacologically targetable (10). The nasal mucosa is also densely innervated by trigeminal sensory fibers. After allergen challenge, patients with AR may show increased CGRP levels in nasal secretions, and intranasal CGRP can induce nasal mucosal blood-flow increase and subjective nasal obstruction (11, 12). These findings support a role for CGRP in local sensory-neuropeptide activation and vascular congestion. However, they do not prove that CGRP determines AR severity, chronicity or treatment resistance. Accordingly, this review distinguishes direct AR evidence from mechanistic hypotheses and from evidence extrapolated from migraine biology or non-nasal barrier tissues. We propose that CGRP may link sensory nerve activation, vascular responses and selected type 2 inflammatory loops in a subgroup of patients with AR. The following sections discuss CGRP biology, clinical evidence, context-dependent immune effects, symptom links, biomarker issues and drug-targeting strategies.

A recent Frontiers in Neurology review has comprehensively summarized neuroimmune communication in AR, including multiple neuropeptides, neuroimmune cell units, TRP channels and MRGPRX2-related pathways (13). The present review is deliberately narrower and mechanistically different. It uses CGRP as the organizing axis and evaluates the hierarchy of nasal-specific evidence and links CGRP most directly to vascular nasal congestion and nasal hyperreactivity, then discusses CGRP receptor antagonism as a testable pharmacological strategy.

### 1.1 Narrative-review search strategy

This article is a narrative review. We searched PubMed, Web of Science and Google Scholar for English-language articles published up to May 2026. Search terms included combinations of “allergic rhinitis”, “nasal mucosa”, “nasal allergen challenge”, “calcitonin gene-related peptide”, “CGRP”, “trigeminal”, “sensory nerve”, “neuropeptide”, “neuroimmune”, “mast cell”, “ILC2”, “dendritic cell”, “eosinophil”, “epithelial alarmin”, “migraine”, “CGRP receptor antagonist”, “gepants” and “zavegepant”. We prioritized human AR studies, nasal challenge studies and nasal mucosal tissue studies. When direct AR evidence was unavailable, airway-barrier, skin, gut or *in vitro* immune studies were used only to define mechanistic hypotheses. These extrapolations are identified in the text and tables.

## 2 CGRP biology in the context of nasal immunity

### 2.1 Isoforms, gene origins and distribution

CGRP mainly includes alpha-CGRP (CGRP-I) and beta-CGRP (CGRP-II). Alpha-CGRP is produced from CALCA by tissue-specific splicing. It is the main subtype in the trigeminal ganglion, dorsal root ganglion and central sensory pathways. Beta-CGRP is encoded by CALCB. It is more common in the enteric nervous system and in some peripheral sensory neurons (9, 14–16). Immunohistochemical work by Baraniuk et al. showed that CGRP-positive sensory fibers in human nasal mucosa are mainly found around vessels in the lamina propria, near submucosal glands and under the epithelium. They are close to SP-positive fibers (17). Many of these fibers arise from the V1/V2 branches of the trigeminal ganglion. This gives CGRP a spatial basis for direct effects on vascular tone, epithelial barrier function and local immune cell activation in the nasal mucosa.

Many CGRP-positive fibers come from the V1 and V2 branches of the trigeminal ganglion. These fibers are found near blood vessels, submucosal glands and the area below the nasal epithelium. This location matters. For example, CGRP released close to small vessels can change vascular tone and contribute to nasal congestion. CGRP released near epithelial and immune-cell areas may also affect barrier activity and local immune responses. In the nasal mucosa, location is not just anatomy; it helps explain how a sensory nerve signal can become a vascular and immune signal.

### 2.2 Receptor complex: CLR, RAMP1 and RCP

Functional CGRP signaling needs the CLR/RAMP1/RCP receptor complex. CLR acts as the G protein-coupled receptor backbone. RAMP1 helps the cell recognize CGRP and move the receptor to the cell membrane. RCP helps pass the signal inside the cell (18, 19). This receptor set works like a matching lock. A cell in the nasal mucosa can respond to locally released CGRP only when the cell has the needed receptor parts.

RAMP1 is the focus of CGRP pharmacology. RAMP1 is not sufficient by itself to verify the function of the CGRP receptor. CGRP can also activate the AMY1 receptor formed by CTR and RAMP1 under some circumstances (20, 21). Therefore, CGRP target cells in the nasal mucosa should be defined by the co-expression of CALCRL, RAMP1, CRCP and CALCR. One indicator is not sufficient.

### 2.3 Release from nasal sensory neurons

CGRP is mainly contained in large dense-core vesicles of trigeminal sensory nerve endings in the nasal mucosa. Nociceptor activation and Ca2+ influx are required for release. TRPV1 and TRPA1 are the two main entry points (22). TRPV1 is a receptor for capsicum, acid and inflammatory agents. TRPA1 is sensitive to cold, oxidative stress products and irritant chemicals (23, 24). Open these channels and then depolarize the nerve endings. CGRP, SP and other neuropeptides are then released.

CGRP is released in the AR microenvironment through a linked cascade of allergen-immune mediator-sensory nerve. Nasal allergen challenge increases CGRP in nasal secretions. Histamine alone does not reliably cause the release of SP and CGRP (11, 25). Therefore, for a good response by CGRP, there needs to be a full-blown allergic inflammation. Only one mast cell mediator is not the cause. CGRP is a neurogenic vasodilator that works locally in the nose after release. Local degradation systems may also affect the effect of neutral endopeptidase (26).

### 2.4 Target cells in the nasal mucosa

The first CGRP target that is expressed in the nasal mucosa is the blood vessels. The first radioligand binding experiments have shown that the 125I-CGRP binding sites in human inferior turbinate mucosa are concentrated in small muscular arteries and arteriolar walls. This is consistent with the physiological effect of CGRP, vasodilation and nasal congestion (17). Uddman et al. also found CGRP receptor-related transcripts in human nasal mucosa. This supports possible CGRP responses in local vessels, epithelium and gland regions (27).

**Figure 1:**
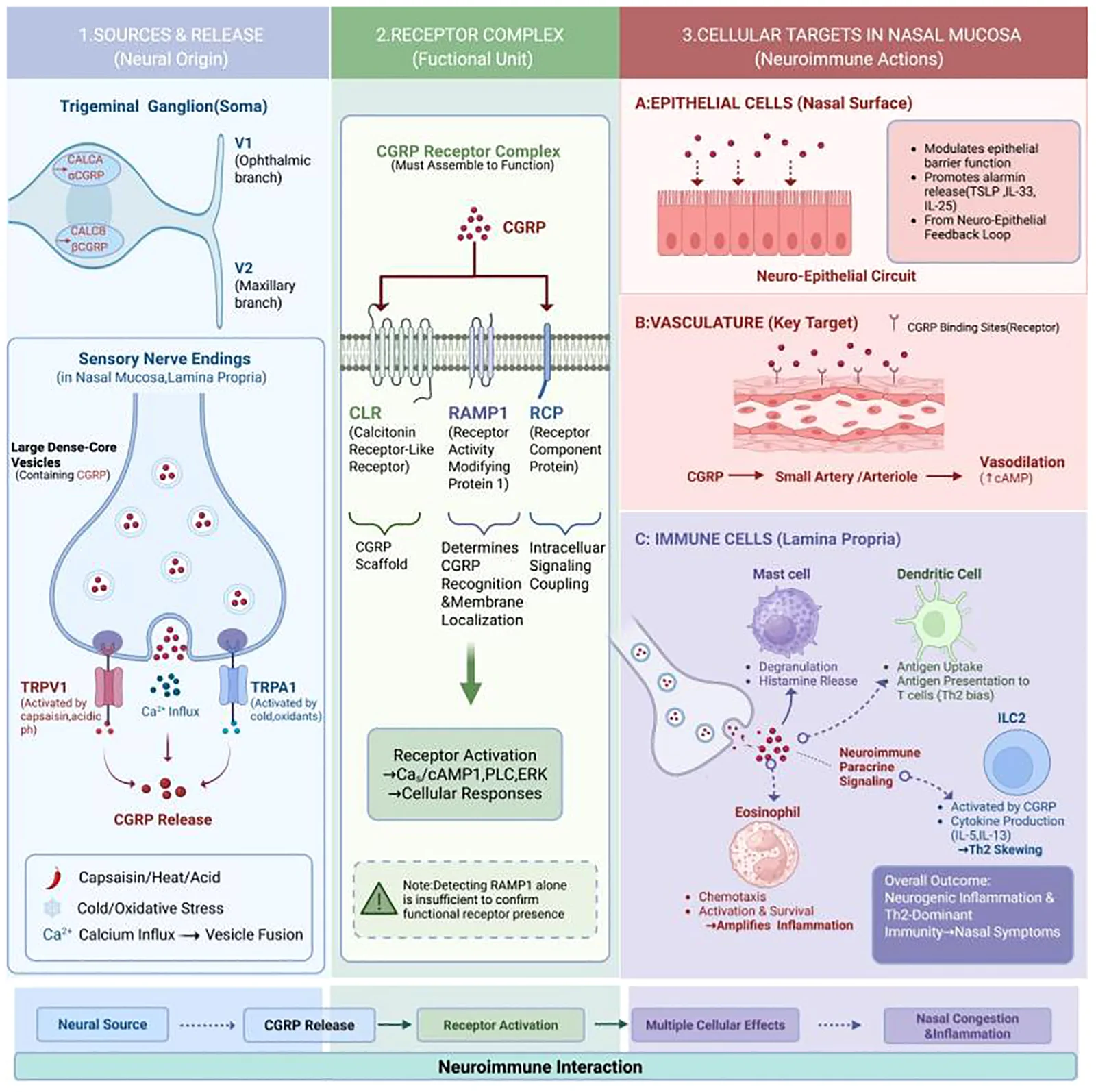
Evidence-layered CGRP-centered neuroimmune network in allergic rhinitis. Solid arrows indicate strong direct or nasal-relevant evidence, dashed arrows indicate moderate or related airway/barrier evidence, and dotted arrows indicate putative or extrapolated mechanisms. The thick CGRP-to-vascular smooth muscle arrow highlights the best-supported nasal effect: CGRP-mediated vasodilation and nasal congestion. ”(Created in https://BioRender.com).

Immune cells form a more complex target group. Direct evidence in nasal mucosa is still limited. Yet barrier immune studies show that CGRP can regulate dendritic cells, Langerhans cells, monocytes/macrophages and T cells (28). In AR nasal mucosa, mast cells, dendritic cells, eosinophils and epithelial cells are close to CGRP-positive nerve fibers. This close location may allow local CGRP release to become a neuroimmune paracrine signal. The spatial relationships between CGRP-positive sensory fibers and these target cells collectively constitute the layered neuroimmune network shown in **Figure 1**.

## 3 Clinical and experimental evidence linking CGRP to AR

### 3.1 CGRP elevation in nasal secretions and tissues

Current clinical evidence can be grouped into three lines. First, nasal allergen challenge can increase SP, CGRP and VIP in nasal secretions. This suggests that sensory neuropeptide pathways and mast cell mediator release are activated together during AR attacks (11, 25). Second, recent studies of naso-ocular neuropeptides show that CGRP can be detected as part of the local neuropeptide network in allergic rhinoconjunctivitis and AR. However, its correlation with VAS scores is less stable than that of SP and VIP (29). Third, tissue studies support neuropeptide network remodeling more than universal CGRP elevation. Several neuropeptide fibers and inflammatory cells change in AR nasal mucosa. Yet CGRP elevation is not consistent across studies (30, 31).

Thus, the clinical role of CGRP in AR should be stated with caution. It is not a mature diagnostic marker. It also cannot represent disease severity on its own. A more reasonable view is that CGRP reflects local sensory nerve activation. It may mark AR subgroups with marked nasal congestion, nasal hyperreactivity or migraine comorbidity.

### 3.2 Temporal dynamics after allergen challenge

Nasal Allergen Challenge (NAC) offers an opportunity for *in vivo* observation of CGRP release. Walker and others found that CGRP increased in the nasal lavage fluid after grass pollen challenge in allergic subjects. The Time Window was extended to 24 hours. Histamine mainly increased in the first 15–60 minutes (25). Mosimann et al. also found that allergen challenge increased SP, CGRP and VIP together. Histamine alone did not show the same neuropeptide profile (11).

This time difference is also a mechanism. CGRP is a vasodilator that can cause vasodilation and nasal congestion. Mucosal congestion and neural hypersensitivity may also occur late in the illness. Research on environmental exposure chambers has rarely included CGRP measurements. Continuous pollen exposure can still maintain mediator release and nasal symptoms (32). Support the continued neurogenic inflammation. Therefore, the changes in CGRP release should not be regarded as simple biochemical variations. They may show that the neurogenic component of AR enters the clinical course.

### 3.3 Symptom induction by intranasal CGRP

Intranasal CGRP challenge also supports the link between symptoms and CGRP. Rangi and others have administered CGRP locally to human nasal mucosa. A larger amount of blood flowed into the nose at that time. Most of the subjects had subjective nasal obstruction. There was no increase in histamine in the nasal exudates in response to this (12). In other words, CGRP does not require mast cell histamine release to cause vascular nasal congestion directly.

However, CGRP is not a typical cause of all nasal symptoms. Guarnaccia and others did not find a significant increase in total protein, albumin or mucoglycoconjugates in nasal lavage fluid with intranasal CGRP. It can be concluded that it will not increase gland secretion or cause plasma leakage directly (33). This negative result is important. It shows that CGRP is not a broad inflammatory mediator. It is more closely linked to vasodilation and mucosal congestion. When SP, histamine, leukotrienes and other mediators are present, CGRP may then amplify rhinorrhea and edema (34).

### 3.4 AR-migraine comorbidity as a mechanistic clue

The comorbidity between AR and migraine provides a clinical clue for a shared trigeminal-CGRP axis. Systematic review and meta-analysis show that patients with AR have a higher risk of migraine (35). A Korean national cohort study also supports a link between AR and later migraine risk (36). Migraine attacks involve the trigeminovascular system and CGRP release. Thus, this comorbidity suggests that nasal mucosal inflammation and migraine may share some sensory nerve sensitization mechanisms (37).

However, comorbidity is not causal proof. Mendelian randomization did not confirm a clear genetic causal relation between AR and migraine (38). Thus, AR-migraine comorbidity should be viewed as a mechanistic clue. It should not be used as evidence that CGRP-targeted treatment can directly improve AR. Still, the shared CGRP axis may support stratified treatment for AR patients with migraine.

### 3.5 Limitations of current clinical evidence

Current clinical data outline a possible role for CGRP in local neurogenic responses in AR, but the evidence chain remains incomplete. Direct studies are mostly small nasal challenge, nasal lavage or physiological provocation studies. Recent patient studies have not consistently shown a linear relationship between CGRP levels and TNSS/VAS scores or overall disease severity (29). Therefore, allergen-induced CGRP elevation should be interpreted as evidence of sensory-neuropeptide pathway activation, not as proof of pathogenic causality. Similarly, intranasal CGRP provocation provides functional evidence for vascular obstruction, but does not establish that CGRP independently drives the full type 2 inflammatory program in AR. More importantly, anti-CGRP agents or CGRP receptor antagonists have not been tested in dedicated randomized controlled trials for AR. CGRP should therefore be viewed as a candidate mechanism and stratification target in AR, not as a validated therapeutic target (9). These findings are systematically summarized with their respective evidence strengths in **Table 1**.

**Table 1:** Clinical and experimental evidence linking CGRP to allergic rhinitis, with evidence strength.

Evidence category	Study/model	Sample or intervention	CGRP-related finding	Interpretation for AR	Key references	Evidence strength
Nasal allergen challenge	Human AR or atopic subjects challenged with allergen	Nasal secretions or nasal lavage fluid	Allergen challenge increased nasal neuropeptides, including CGRP, often together with SP and VIP	Direct clinical evidence that allergen exposure can activate sensory neuropeptide pathways in AR	(11, 25)	Moderate: direct human nasal evidence, although largely from small challenge studies
Temporal release dynamics	Nasal challenge with grass/ryegrass antigen	Serial nasal lavage after challenge	CGRP increased after allergen exposure, with a time window extending beyond the early histamine-dominant phase	Supports a role for CGRP in both early vascular responses and later neurogenic sensitization	(25)	Moderate: direct human nasal evidence with defined temporal sampling
Nasal secretion and ocular-neuropeptide network	Patients with allergic rhinoconjunctivitis, AR or allergic conjunctivitis	Nasal secretions and tears	CGRP was detectable as part of a local naso-ocular neuropeptide network, although symptom correlation was weaker than SP/VIP	CGRP is better viewed as a marker of local neuroimmune activation than as a standalone severity biomarker	(29)	Limited to moderate: human data with heterogeneous methods and inconsistent symptom correlations
Nasal mucosal tissue	Human AR and control nasal mucosa	Immunohistochemistry or tissue neuropeptide assessment	AR showed neuropeptide-fiber and inflammatory-cell remodeling, but CGRP elevation was not consistent across studies	Tissue data support neural remodeling in AR, while cautioning against treating CGRP as universally elevated	(30, 31)	Limited to moderate: human tissue evidence with inconsistent CGRP elevation
Intranasal CGRP administration	Human nasal provocation	Topical nasal CGRP	CGRP increased nasal mucosal blood flow and induced subjective nasal obstruction without parallel histamine release	Provides functional evidence that CGRP can directly drive vascular nasal congestion	(12)	Strong for vascular congestion; limited for broader inflammation
Secretory/extravasation response	Human nasal CGRP provocation	Nasal lavage proteins, albumin and mucoglycoconjugates	Intranasal CGRP did not robustly increase glandular secretion or plasma protein leakage	CGRP is not a broad secretagogue; its clearest nasal effect is vascular	(33)	Limited: negative provocation data help define symptom boundaries
AR-migraine comorbidity	Population-based and meta-analytic studies	Epidemiological association	AR was associated with increased migraine risk, but genetic causal evidence remains limited	Shared trigeminal-CGRP biology may identify a patient subgroup, not prove AR treatment efficacy	(35–38)	Hypothesis-generating for AR therapy
Current evidence gap	Pharmacological intervention in AR	Anti-CGRP mAbs, gepants or intranasal CGRP receptor antagonists	No AR-specific randomized controlled trial is currently established in the manuscript evidence base	CGRP remains a mechanistic candidate and stratification target, not a validated AR therapy	(9, 29)	Hypothesis-generating: targetability shown in migraine, but efficacy unproven in AR

## 4 Cellular and molecular mechanisms of CGRP-mediated amplification

Direct mechanistic studies in AR are limited. This section therefore uses three evidence levels: direct AR evidence, airway-related evidence and extrapolated evidence. This grading is necessary. CGRP effects depend strongly on tissue setting, cell type and inflammatory stage.

### 4.1 Evidence hierarchy and interpretative framework

To avoid overstating the evidence, this review uses three evidence levels. First, direct AR/nasal evidence includes patient nasal secretions, nasal mucosal tissue, nasal allergen challenge and intranasal CGRP provocation. Second, related airway-barrier evidence includes asthma, chronic rhinosinusitis, nasal hyperreactivity and lower-airway type 2 inflammation (39). These conditions share epithelial, sensory-neural and type 2 immune features with AR, but differ anatomically and clinically. Third, extrapolated evidence includes skin, gut, lung parenchyma and *in vitro* immune-cell studies. These studies are useful for hypothesis generation, but their mechanisms cannot be assumed to operate identically in human nasal mucosa. Direct validation requires evidence of receptor co-expression, spatial proximity and disease-context relevance.

### 4.2 Context-dependent and potentially anti-inflammatory CGRP effects

CGRP should not be regarded as a uniformly pro-inflammatory mediator. In several immune settings, CGRP can suppress or reshape dendritic-cell activation, reduce selected TLR-driven cytokine responses through cAMP/PKA-related pathways and modulate T-cell polarization or effector function (28, 40–42). ILC2 studies also indicate bidirectional effects. CGRP can restrain some IL-13-associated responses in certain contexts, while supporting IL-5-related or airway-neuroendocrine type 2 programs in others (43–45). These observations suggest that CGRP functions as an immunoregulatory signal whose net effect depends on timing, tissue compartment, receptor expression and the epithelial alarmin milieu. In AR, we therefore use the term “neuroimmune amplifier” in a restricted sense. The strongest direct support is for amplification of vascular congestion after sensory nerve activation. Immune amplification through mast cells, ILC2s, dendritic cells, eosinophils and epithelial alarmins remains plausible, but requires direct validation in human nasal mucosa.

### 4.3 CGRP-mast cell axis: priming the immediate response

Mast cells are core effector cells in the early phase of AR. After re-exposure to allergen, they rapidly release histamine, tryptase, PGD2 and leukotrienes (46, 47). CGRP-positive sensory fibers are close to mast cells under the nasal epithelium and around vessels. This provides a structural basis for local neuroimmune units (17, 48). Direct evidence that CGRP enhances mast cell degranulation in AR nasal mucosa is limited. Still, barrier tissue studies show that house dust mite proteases can activate nociceptive neurons and amplify type 2 inflammation through a neuropeptide-mast cell axis (49). In AR, the key point is not that CGRP replaces the IgE pathway. Rather, mast cell tryptase may induce CGRP and SP release through PAR2. This may maintain vasodilation and neurogenic inflammation (50). Thus, the mast-cell axis should be interpreted as a plausible bidirectional nerve-mast-cell feedback loop in AR. This interpretation is supported by nasal anatomy and extrapolated barrier-tissue mechanisms, rather than by direct evidence that CGRP enhances IgE-mediated degranulation in human nasal mucosa.

### 4.4 CGRP-ILC2 axis: context-dependent type 2 modulation

ILC2s link epithelial alarmins to type 2 inflammation. After stimulation by TSLP, IL-25 and IL-33, they produce IL-5 and IL-13 (51, 52). These cytokines support eosinophil recruitment, mucus responses and chronic inflammation (53, 54). The effect of CGRP on ILC2s is not one-way. Some studies show that CGRP limits ILC2 growth and IL-13 production, while keeping or increasing IL-5-related responses. Other airway models suggest that CGRP from pulmonary neuroendocrine cells can amplify ILC2-driven type 2 inflammation (43–45, 55, 56). Thus, in AR nasal mucosa, CGRP may reshape the quality of the ILC2 response. It should not be treated as a fixed pro-inflammatory or anti-inflammatory signal. Accordingly, CGRP may reshape ILC2 output rather than simply intensify type 2 inflammation. Direct nasal ILC2 studies are needed before this pathway can be assigned a definite pathogenic role in AR.

### 4.5 CGRP-dendritic cell axis: Th2 skewing during sensitization

Dendritic cells (DCs) are key initiating cells during AR sensitization. The idea that CGRP regulates antigen-presenting cells first came from Langerhans cell studies. Later studies showed that CGRP can suppress DC inflammatory responses to TLR stimulation through cAMP/PKA-related pathways and can affect T cell differentiation (40–42). In AR, this axis suggests a possible sequence. Allergen or environmental stimuli activate nasal sensory nerves and release CGRP. Nearby DCs may then support Th2 bias in an epithelial setting rich in TSLP, IL-33 and IL-25 (57). Single-cell maps of nasal mucosa have shown changes in antigen-presenting cells and epithelial cell states in AR. The next key step is to confirm whether relevant DC subsets have functional CGRP receptor complexes (58). This mechanism remains hypothesis-generating for AR because most evidence comes from skin or *in vitro* antigen-presenting-cell studies. Nasal DC subsets should be tested for functional CLR/RAMP1/RCP expression and CGRP responsiveness.

### 4.6 CGRP-eosinophil axis: sustaining late-phase inflammation

Eosinophils are important effector cells in late-phase AR and chronic inflammation. Their recruitment, survival and degranulation are controlled by IL-5, IL-13 and eotaxin-family chemokines (59, 60). *In vitro* studies show that CGRP itself is not a strong chemoattractant. Yet it can increase eosinophil chemotactic responses to PAF and LTB4 in allergic subjects. Several neuropeptides, including CGRP, can also induce human eosinophil migration (61, 62). Thus, CGRP may act more as a priming factor in the late phase. It may not directly drive migration, but it can raise eosinophil sensitivity to other inflammatory signals (63). The eosinophil axis is best viewed as a late-phase priming hypothesis, not as evidence that CGRP is a dominant eosinophil chemoattractant in AR.

### 4.7 CGRP-epithelial alarmin axis: closing the neuron-epithelium loop

The nasal epithelium is both a physical barrier and an immune-active structure. It is one of the first sites to sense allergens, pollutants and damage in AR. Barrier injury promotes allergen entry into the lamina propria. It also induces TSLP, IL-33 and IL-25 release. These signals then activate DCs, ILC2s, mast cells and eosinophils (64–67). CGRP may enter this neuron-epithelium-immune loop, but direct AR evidence is still limited. Airway and other barrier tissue studies suggest that CGRP can be released by neuroendocrine or sensory nerve-related structures. It can regulate ILC2 inflammation, epithelial secretion and barrier protection (45, 68). In AR, a cautious model is more appropriate. Allergens and inflammatory mediators activate sensory nerves and release CGRP. CGRP then works through vessels, epithelium and immune cells to amplify local type 2 inflammation. Epithelial alarmins further activate ILC2s and DCs. This closes a positive feedback loop. This neuron-epithelium-immune loop is biologically plausible, but direct evidence that CGRP controls epithelial alarmin release in human AR remains limited.

### 4.8 Integrated amplification model

**Figure 2:**
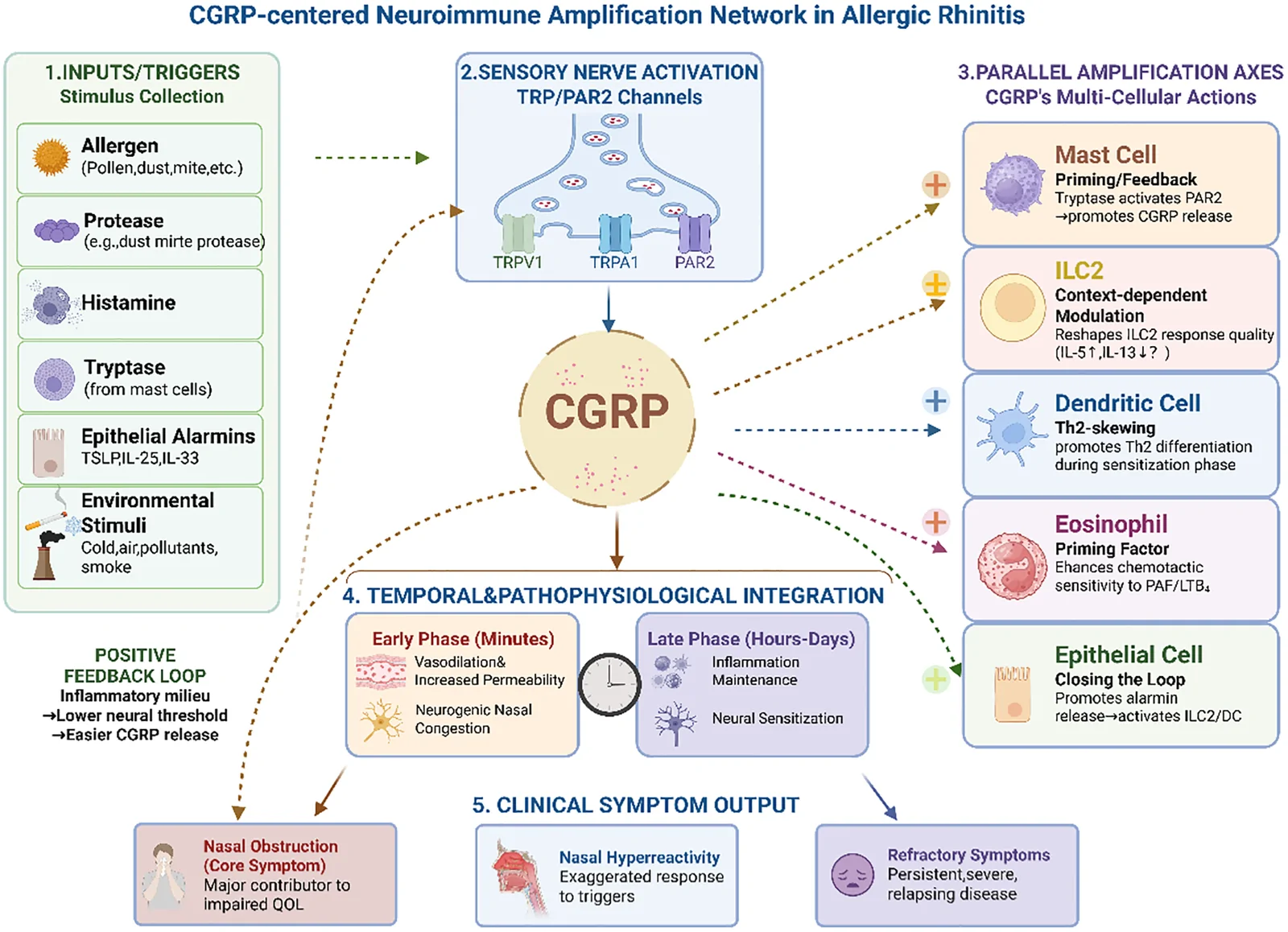
Proposed CGRP-centered neuroimmune amplification network in allergic rhinitis. Allergens, proteases and epithelial alarmins may activate TRPV1/TRPA1/PAR2-related sensory pathways and promote CGRP release. CGRP may then amplify vascular congestion, immune-cell crosstalk and neural sensitization. The model is intended to guide testable endotype and intervention studies. (Created in https://BioRender.com).

Taken together, CGRP is not an effector molecule of a single pathway in AR. It is a cross-cell and cross-phase amplification node. This integrated model of CGRP as a cross-cell and cross-phase amplification node is schematically summarized in **Figure 2**. The proposed effects of CGRP on key cellular and structural targets in AR are detailed in **Table 2**. Allergens, proteases, histamine, tryptase, epithelial alarmins and environmental irritants can activate TRPV1/TRPA1/PAR2-related sensory nerves and promote CGRP release (72). Released CGRP rapidly induces vasodilation. It may also enhance AR inflammation and symptoms through mast cell-nerve feedback, context-dependent ILC2 regulation, DC-Th2 skewing, eosinophil priming and the epithelial alarmin loop. This model explains why CGRP may not correlate linearly with total symptom scores. It may instead mark an AR endotype with marked nasal congestion, nasal hyperreactivity or migraine comorbidity.

**Table 2:** Proposed effects of CGRP on key immune and structural cells in allergic rhinitis, separated by evidence source.

Target cell or structure	Receptor/response context	Proposed CGRP effect	Mechanistic implication in AR	Evidence strength for AR	Key references
Vascular smooth muscle and vascular wall cells	CGRP binding sites enriched around small arteries and muscular arterioles; CLR/RAMP1/RCP signaling activates cAMP pathways	Potent vasodilation and sustained nasal mucosal blood-flow increase	Converts sensory nerve activation into vascular nasal congestion and turbinate engorgement	Strongest AR-relevant evidence: human nasal tissue binding and nasal provocation studies	(12, 17, 69)
Mast cells	Anatomical proximity to CGRP-positive sensory fibers; bidirectional mast cell-neuron signaling	May amplify immediate responses through neuropeptide-mast cell feedback; mast-cell tryptase can activate PAR2 on sensory nerves and promote neuropeptide release	Reinforces early-phase symptoms and sustains neurogenic inflammation, especially nasal congestion	Moderate: nasal anatomical proximity plus extrapolated barrier-tissue mechanisms	(17, 48–50)
ILC2	ILC2s respond to epithelial alarmins and may express neuropeptide-responsive programs	Context-dependent modulation of IL-5/IL-13 output; CGRP can constrain or amplify ILC2 responses depending on tissue setting	Shapes the quality of type 2 inflammation rather than acting as a fixed pro-inflammatory signal	Limited for AR: mechanistic support mainly from airway and gut models	(43–45, 53, 54)
Dendritic cells	CGRP can regulate antigen-presenting cell function through cAMP/PKA-related pathways	Suppression or reshaping of antigen presentation and inflammatory cytokine responses; possible support of Th2-skewing under epithelial alarmin-rich conditions	May influence allergen sensitization and local Th2 bias	Hypothesis-generating for AR: mainly skin and *in vitro* DC studies	(40–42, 58)
Eosinophils	CGRP is not a dominant chemoattractant but can prime eosinophil responsiveness to other mediators	Enhances eosinophil chemotactic sensitivity to PAF/LTB4 and supports neuropeptide-associated migration	May help maintain late-phase inflammation rather than initiate eosinophil recruitment	Limited: mainly *in vitro* and extrapolated evidence	(59–63)
Nasal epithelium and epithelial alarmin axis	Nasal epithelial barrier disruption promotes TSLP, IL-33 and IL-25 release; CGRP may interact with epithelial-neuroimmune loops	Potential amplification of epithelial alarmin-ILC2/DC signaling and barrier-associated inflammatory feedback	Closes the neuron-epithelium-immune loop that sustains type 2 inflammation and hypersensitivity	Hypothesis-generating: direct AR evidence remains limited	(45, 64–68)
Sensory neurons	TRPV1/TRPA1/PAR2-related activation promotes CGRP release; inflammatory mediators lower activation threshold	Autocrine/paracrine reinforcement of neurogenic sensitization	Explains persistent hypersensitivity to cold air, odors, smoke and low-level irritants	Moderate: supported by AR hyperreactivity and neuroimmune literature, although CGRP-specific intervention evidence is lacking	(23, 24, 50, 70, 71)

## 5 Clinical stratification and CGRP-linked AR endotypes

The key role of CGRP in symptoms is not that it explains all AR features alone. Its main role is to convert neural activation into vascular nasal congestion. It may also amplify edema and reflex sensitization when other inflammatory mediators are present. Nasal congestion is the clearest example. CGRP is a strong vasodilator peptide. It can activate Gs-cAMP-PKA signaling through the CLR/RAMP1/RCP receptor and reduce vascular smooth muscle tone (9, 69). In human nasal mucosa, CGRP binding sites are concentrated in small arteries and muscular arterioles. Intranasal CGRP can also induce sustained nasal mucosal blood-flow increase and subjective nasal obstruction (12, 17). Thus, the most direct and testable contribution of CGRP to AR symptoms is vascular nasal congestion after sensory nerve activation.

By contrast, rhinorrhea, sneezing and nasal itching cannot be explained by CGRP alone. Intranasal CGRP challenge did not clearly increase nasal lavage proteins or mucoglycoconjugates. This suggests that CGRP is not a main glandular secretagogue (33). However, CGRP may enhance edema caused by permeability mediators such as histamine, bradykinin, PAF, leukotrienes and SP through sustained vasodilation. Sensory nerve stimulation can also promote plasma extravasation in AR nasal mucosa (34, 73). Sneezing and itching depend more on trigeminal sensory reflexes, TRPV1/TRPA1 channels and mediator-driven lowering of neural thresholds (74, 75). In this framework, CGRP is not the initiating factor for sneezing or rhinorrhea. It may prolong local sensitization and make symptoms easier to trigger by non-specific stimuli.

This also explains why the relation between CGRP and disease severity may be non-linear. In some patients with AR, symptoms are not limited to short attacks after allergen exposure. They show nasal hyperreactivity to cold air, odors, smoke and temperature change (70, 71). Repeated inflammation can lower the threshold of TRPV1/TRPA1-positive sensory nerves. Low-level stimuli can then induce neuropeptide release. After release, CGRP enhances vasodilation and the local inflammatory setting. The inflammatory setting further sensitizes sensory nerves. This forms a cycle of allergic inflammation, neuropeptide release, vascular or immune amplification and neural sensitization. The most useful clinical role of CGRP may not be to explain all AR patients. It may help identify subgroups with marked nasal congestion, strong nasal hyperreactivity or incomplete response to standard treatment (11, 12, 29).

The proposed “CGRP-high” AR endotype should be treated as a research construct rather than an established clinical category. It would need to be identified through repeated nasal-secretion CGRP measurements, a congestion-dominant symptom pattern, objective nasal airflow reduction, nasal hyperreactivity to non-specific stimuli and, possibly, migraine comorbidity. A single high CGRP value after allergen exposure is unlikely to be sufficient. CGRP release may vary with allergen load, season, sampling time, local degradation, recent medication use and epithelial injury. This endotype may overlap with severe or persistent AR, but should not be defined only by ARIA severity or duration. Within current ARIA categories, CGRP-high status is better considered an additional treatable trait. It may occur in seasonal AR, perennial AR, severe AR or mixed rhinitis phenotypes, particularly when congestion and neural hyperreactivity are prominent.

The mechanistic synthesis above is most useful clinically if it defines a subgroup rather than a universal AR pathway. The most defensible CGRP-linked endotype would be congestion-dominant and neurogenic. Candidate features include persistent or allergen-induced nasal obstruction, objective airflow limitation, nasal hyperreactivity to cold air, odors, smoke or temperature change, elevated nasal secretion CGRP during challenge or exposure, and migraine comorbidity. This profile is consistent with the strongest nasal evidence for CGRP, namely vascular effects and sensory-neuropeptide activation, while avoiding the unsupported claim that CGRP explains all AR symptoms.

This endotype should be separated from broader type 2-high AR. Patients with prominent sneezing, itching and rhinorrhea may still be driven mainly by IgE-mast cell activation, histamine, leukotrienes, epithelial alarmins and eosinophilic inflammation. In those patients, CGRP may contribute to vascular amplification or sensory threshold lowering, but it is unlikely to be the dominant therapeutic node. Clinically, this distinction argues for CGRP-directed studies to enrich for congestion, hyperreactivity and CGRP-high biology, rather than enrolling unselected AR populations.

A practical stratification scheme should therefore combine symptoms, physiology and biomarkers. First, patients can be screened for congestion-dominant disease, nasal hyperreactivity and incomplete response to standard anti-inflammatory treatment. Second, objective measures such as nasal airflow, acoustic rhinometry or rhinomanometry can be paired with nasal secretion CGRP before and after allergen or non-specific challenge. Third, tissue-based studies should test whether CGRP-high patients show CLR/RAMP1/RCP expression in vascular, epithelial and immune-cell niches. This approach turns the CGRP hypothesis into a falsifiable endotype model and provides a rational entry point for proof-of-concept trials.

## 6 Pharmacological targeting and translational roadmap

### 6.1 Rationale for CGRP targeting in AR

If CGRP mainly amplifies vascular congestion and neurogenic sensitization, then CGRP blockade should first be tested in AR subgroups enriched for those features, not in unselected AR populations. Receptor pharmacology, nasal provocation studies and migraine therapeutics together support biological plausibility (76). However, no dedicated randomized controlled trial has established efficacy of anti-CGRP monoclonal antibodies, CGRP ligand antagonists or CGRP receptor antagonists in AR. CGRP targeting in AR should therefore be defined as a drug-repositioning and endotype-testing hypothesis, not as a clinically recommended treatment.

### 6.2 Monoclonal antibodies and oral gepants: useful precedent, uncertain nasal efficacy

Migraine studies have shown that the CGRP pathway can be blocked by monoclonal antibodies and small-molecule gepants (10, 77–86). This experience is relevant to AR only as proof of pharmacological targetability. It should not be interpreted as evidence that CGRP blockade improves allergic nasal inflammation. Migraine and AR are biologically different diseases: migraine is dominated by trigeminovascular pain sensitization, whereas AR is a mucosal barrier disease involving allergen-specific IgE, mast cells, epithelial alarmins, ILC2s, eosinophils, vascular responses and sensory reflexes. Systemic monoclonal antibodies are target-specific and long acting, but it is unclear whether they reach effective local levels in the nasal mucosa, whether they alter mucosal immune-cell function, or whether long-term blockade affects barrier defense. Oral gepants are more flexible and reversible, but nasal receptor occupancy and efficacy against IgE-driven sneezing, rhinorrhea and late-phase inflammation are unknown. Thus, mAbs and oral gepants provide a pharmacological precedent; they do not provide treatment evidence for AR.

### 6.3 Intranasal zavegepant: the most translatable candidate

Intranasal zavegepant is a rational candidate for early mechanistic testing because it targets the CGRP receptor and its route of delivery matches the target organ in AR. Zavegepant has shown efficacy in acute migraine treatment and was approved in the United States in 2023 for acute migraine treatment in adults (87–89). In AR, however, the relevant question is not whether zavegepant treats migraine. The key issue is whether local nasal CGRP receptor blockade can reduce CGRP-mediated vascular congestion or neural hyperreactivity under allergic inflammatory conditions.

Zavegepant should therefore be framed as a proof-of-concept tool rather than as a ready AR therapy. Suitable early studies would enroll AR patients with marked nasal congestion, clear nasal hyperreactivity, reproducibly high nasal-secretion CGRP or migraine comorbidity. Endpoints should be AR-specific and include congestion VAS, objective nasal airflow, nasal allergen-challenge responses, CGRP/SP changes in nasal secretions, non-specific stimulus thresholds and local tolerability. Migraine endpoints should not be transferred directly to AR.

### 6.4 Indirect strategies: TRPV1/TRPA1 modulation

Another strategy is to reduce CGRP release by acting upstream on sensory nerve activation. TRPV1 and TRPA1 help the nasal mucosa sense capsaicin, cold air, oxidative stress and irritant gases. Their activation can promote CGRP and SP release (23, 24, 70). Intranasal capsaicin can desensitize sensory nerves after repeated activation. It has shown some benefit in non-allergic rhinitis (90, 91). However, TRP channels are widely involved in temperature sensing and mucosal protection. Systemic antagonists raise safety and specificity concerns (92). Therefore, this strategy may fit AR subgroups with strong neural hyperreactivity or non-specific stimulus sensitivity.

### 6.5 Barriers, safety and proof-of-concept roadmap

Migraine mainly involves the trigeminovascular system and pain sensitization. CGRP is a central disease and treatment node in that setting. AR is different. It is a mucosal inflammatory disease driven by the nasal epithelial barrier, IgE-FceRI, mast cells, ILC2s, eosinophils, vascular responses and sensory nerve reflexes (1, 9). Blocking CGRP may improve nasal congestion or neural hyperreactivity. It may not be enough to suppress allergen-specific IgE responses or late-phase inflammation. Safety must also be considered early. CGRP is involved in vascular homeostasis, tissue repair, barrier protection and host defense. Long-term or systemic blockade should be assessed for cardiovascular risk, blood pressure change, constipation, pregnancy and infection risk (9, 93–95).

Future studies should start with small mechanism-focused proof-of-concept trials. They should not move directly to large efficacy trials. A reasonable design would enroll AR patients with marked nasal congestion or nasal hyperreactivity. Baseline nasal secretion CGRP should be measured. A local CGRP receptor antagonist could then be tested for a short period. Main endpoints may include nasal congestion VAS, nasal airflow, CGRP/SP changes after allergen challenge and thresholds for non-specific stimuli. If baseline CGRP predicts the response to zavegepant, a CGRP-high AR endotype would have real translational value. At the same time, spatial transcriptomics and single-cell maps should define contact sites between CGRP-positive nerves and mast cells, ILC2s, DCs, eosinophils and epithelial subsets (58, 96–99). In this framework, CGRP targeting is more likely to supplement intranasal corticosteroids, antihistamines, AIT or dupilumab than to replace them.

### 6.6 Biomarker and feasibility issues for nasal CGRP measurement

Nasal CGRP measurement is promising but remains investigational. Before it can be used for patient selection, several technical and biological issues must be resolved. Sampling methods, including nasal lavage, filter-paper absorption and direct secretion collection, may yield different peptide concentrations because dilution, mucosal contact time and local secretion volume differ. Assay reproducibility, inter-laboratory calibration and lower limits of detection also require standardization. CGRP may vary with allergen season, recent exposure, time after nasal challenge, medication use, comorbid migraine, infection, epithelial injury and local peptidase activity. Pre-analytical handling is equally important, because peptide stability may be affected by collection medium, temperature, processing delay and freeze-thaw cycles. Therefore, a clinically useful CGRP biomarker will require standardized sampling, paired symptom and airflow measurements, repeated testing and prospectively defined thresholds. Until such validation is available, nasal CGRP should be considered a mechanistic and stratification biomarker for research rather than a routine diagnostic test.

## 7 Conclusions

The main value of CGRP in allergic rhinitis (AR) is not that it acts as an independent pro-inflammatory mediator. It may act as a neuroimmune amplifier. It links sensory nerve activation, vasodilation and type 2 inflammation into a continuous disease network. Current evidence suggests that CGRP may take part in early-phase responses, late-phase responses and persistent hyperreactivity. However, direct clinical evidence is still limited to small challenge studies and extrapolated data. It is far below the level needed to support routine clinical use. The success of migraine therapy only shows pharmacological feasibility. It does not mean that the same treatment logic can be directly copied to AR. AR is a multi-cell and multi-pathway disease. Future work should first define a CGRP-high endotype. It should then use proof-of-concept trials of intranasal receptor antagonists. These trials should test whether local CGRP levels, nasal congestion phenotype and treatment response are stably linked. Only then can this hypothesis move from mechanism to precise intervention.
